# NMDA Receptor Regulation by Calmodulin and α-Actinin-1

**DOI:** 10.3390/biom16081146

**Published:** 2026-08-07

**Authors:** Aritra Bej, Johannes W. Hell, James B. Ames

**Affiliations:** 1 Department of Pharmacology, University of California, Davis, CA 95616, USA; abej@health.ucdavis.edu (A.B.); jwhell@ucdavis.edu (J.W.H.); 2 Department of Chemistry, University of California, Davis, CA 95616, USA

**Keywords:** NMDAR, GluN1, GluN2A, GluN2B, CDD, calmodulin, α-actinin

## Abstract

N-methyl-D-aspartate (NMDA) receptors (NMDARs) are Ca^2+^-permeable ionotropic glutamate receptors in the brain that have critical roles in learning, memory, neural development, and synaptic plasticity. NMDARs are heterotetrameric Ca^2+^ channels, which open upon binding to the neurotransmitters, glutamate and glycine. Channel opening causes Ca^2+^ influx that activates a range of Ca^2+^-dependent cellular processes, including activation of Ca^2+^-dependent enzymes, which mediates various forms of synaptic plasticity. Prolonged channel opening elevates the intracellular Ca^2+^ level to a cytotoxic concentration. To maintain Ca^2+^ homeostasis, NMDAR channel activity is finely regulated by α-actinin (ACTN), which promotes channel opening by reducing the closed time, and by calmodulin (CaM), which promotes Ca^2+^-dependent channel desensitization (CDD). Defects in the regulation of NMDAR function are associated with a spectrum of neurological diseases. In this review, we integrate cryo-EM structures of NMDARs, NMR structures of the NMDAR cytosolic C0 domain of the GluN1 and GluN2A subunits bound to Ca^2+^-bound CaM (Ca^2+^-CaM), and various structures of α-actinin-1 (ACTN1) to construct structural models of NMDAR in the open channel state bound to Ca^2+^-free ACTN1 and the agonist-bound, desensitized channel state bound to Ca^2+^-CaM. These structural models provide insights into the Ca^2+^-dependent conformational changes that promote CDD.

## 1. Overview of NMDA Receptors

**Figure 1:**
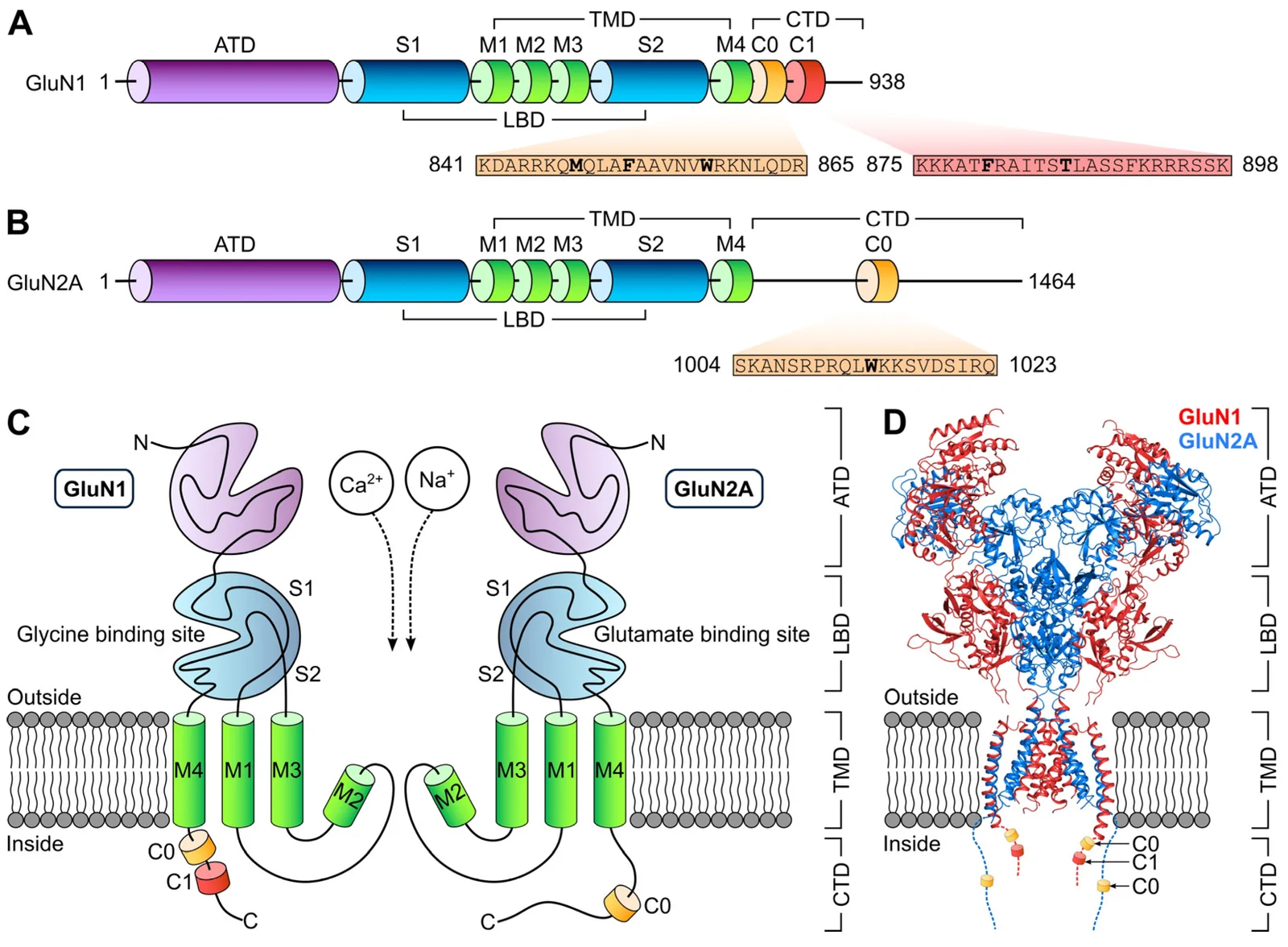
Domain organization in the NMDA receptor. Linear representations of (**A**) GluN1 and (**B**) GluN2A domain architecture. Each subunit consists of four domains: the extracellular amino-terminal domain (ATD) and ligand binding domain (LBD) where glycine and glutamate bind to the GluN1 and GluN2 subunits, respectively, the transmembrane domain (TMD), and the intracellular carboxy-terminal domain (CTD). The two CaM binding sites (C0 and C1) in GluN1 and the single CaM binding site (C0) in GluN2A are indicated by boxes. Critical residues involved in CaM interactions are highlighted in bold. (**C**) Schematic representation of the assembly and modular organization of the NMDA receptor. Dashed arrows indicate the influx of Ca^2+^ and Na^+^. (**D**) Cryo-EM structure of the NMDA receptor (PDB ID: 6IRA [15]). GluN1 and GluN2A subunits are colored red and blue, respectively. The CTD was truncated and not determined in the experimental structures. Intracellular CaM binding sites (GluN1-C0, GluN1-C1, and GluN2A-C0) are represented as cylinder and labeled.

N-methyl-D-aspartate (NMDA) receptors (NMDARs) are ligand-gated and Ca^2+^-permeable ion channels expressed throughout the central nervous system and are most often localized at the postsynaptic membrane in the brain. The activation of NMDARs results in excitatory currents that underlie neural development, synaptic plasticity, learning, and memory [1,2,3,4,5,6]. Mutations in NMDARs cause severe neuropsychiatric disorders and have been implicated in chronic pain syndromes and neurodegenerative diseases [7]. NMDARs assemble as heterotetrameric ion channels (Figure 1) composed of two obligatory GluN1 subunits that bind glycine (or D-serine) and two subunits that can be any combination of glutamate-binding GluN2 or glycine-binding GluN3 subunits. RNA splicing produces eight distinct GluN1 [8], four GluN2(A-D), and two GluN3(A-B) subunits [9]. The four GluN2 subtypes are differentially expressed in nervous tissue [10,11]. The NMDAR subunits interact with numerous auxiliary binding proteins [12,13,14] that are necessary for proper trafficking, targeting, turnover, and regulation of NMDARs. In this review, we will focus on NMDAR interactions with the intracellular proteins, calmodulin (CaM) and α-actinin-1 (ACTN1).

NMDAR subunits contain a modular domain architecture (Figure 1A–C), including an extracellular amino-terminal domain (ATD, purple in Figure 1A–C), ligand binding domain (LBD, cyan in Figure 1A–C), a central transmembrane domain (TMD, green in Figure 1A–C), and an intracellular carboxy-terminal domain (CTD, yellow and red in Figure 1A–C) [16,17]. The LBD harbors binding sites for the obligatory co-agonists, glutamate and glycine. The TMD consists of three transmembrane helices (M1, M3, and M4) and a re-entry loop that includes a short M2 helix (Figure 1C). The CTD contains helical segments (called C0 and C1 in Figure 1A–C) that bind to CaM [12,18,19] and ACTN [20,21,22]. Activation of NMDARs upon binding of agonist glutamate and co-agonist glycine opens the channels and causes an increase in intracellular Ca^2+^ concentration, thus initiating a wide range of Ca^2+^-dependent downstream signaling pathways.

**Figure 2:**
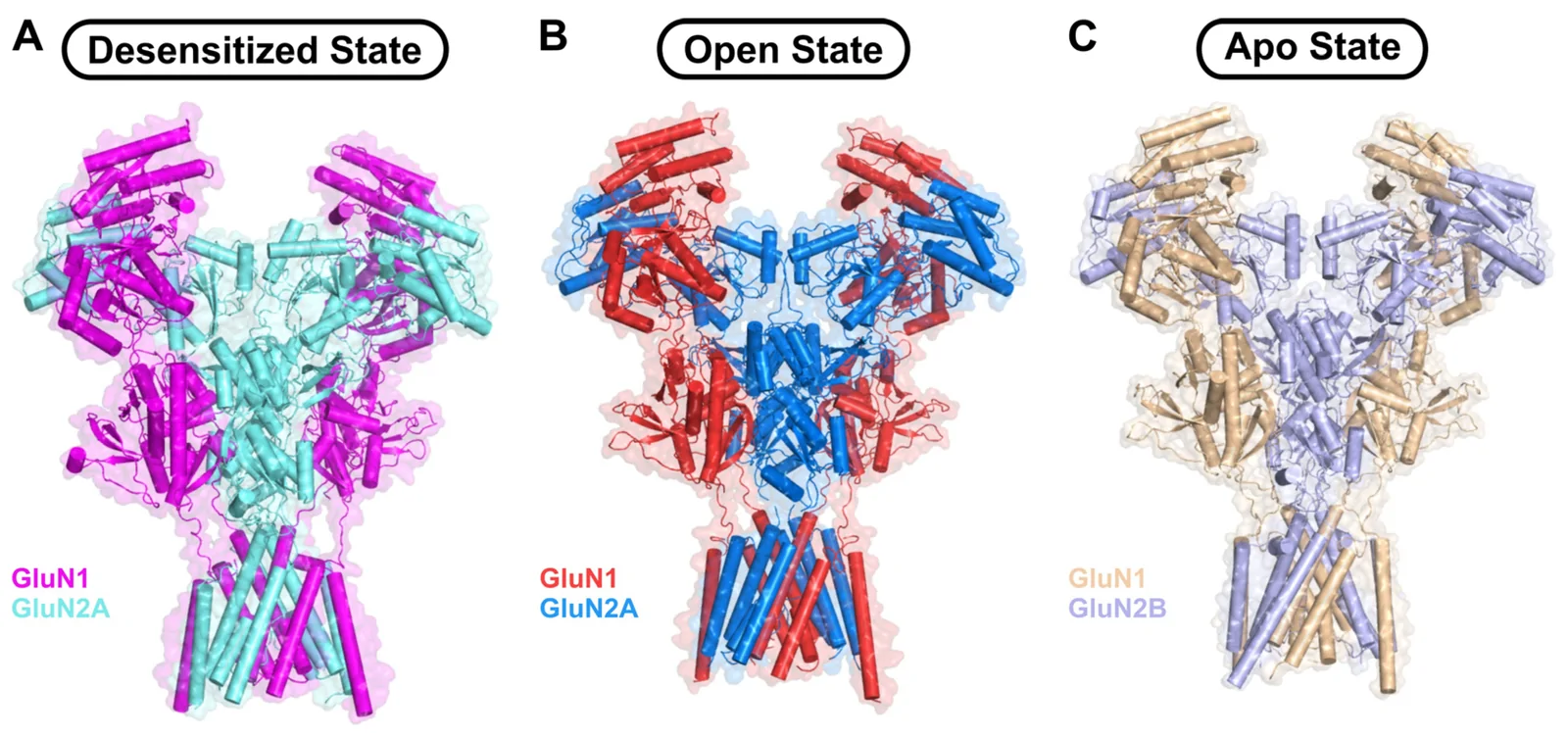
Structures of the NMDARs in different conformational states. Cryo-EM structures of GluN1-GluN2A NMDA receptor in the ligand-bound (**A**) desensitized state (PDB ID: 9UNN [28]), (**B**) open state (PDB ID: 7EOS [30]), and (**C**) GluN1-GluN2B NMDAR in apo conformation (PDB ID: 9ARG [31]).

Three-dimensional structures of NMDARs (Figure 1D and Figure 2A–C) have been determined by x-ray crystallography [16,17,23,24] and cryo-electron microscopy (cryo-EM) [25,26,27,28,29]. The structures show detailed inter-subunit interactions between the ATD and LBD and between the LBD and TMD. A recent cryo-EM structure of the native GluN1-GluN2A NMDAR represents the heterotetrameric channel in a ligand-bound desensitized state (Figure 2A) in which glutamate and glycine are bound to the LBD and the channel pore is closed [28]. Other cryo-EM structures captured the agonist-bound NMDAR in the open state (Figure 2B) with the channel pore fully open [25,30]. A cryo-EM structure is also known for GluN1-GluN2B NMDAR in the apo state (Figure 2C), in which no ligand is bound to the LBD and the channel pore is closed [31]. The structure of the CTD is missing in all known structures, because the NMDAR samples in the structural studies either lacked the CTD or the CTD was dynamically disordered in the native NMDAR structures [28]. Therefore, the dynamical conformational changes in the CTD that underlie channel modulation by CaM and ACTN are not resolved by x-ray crystallography or cryo-EM. In this review, we use computational modeling to predict structural models of the NMDAR with a structured CTD bound to Ca^2+^-CaM or ACTN1 in the desensitized or open channel states, respectively (see Section 5).

## 2. Ca^2+^-Dependent Desensitization of NMDARs by CaM

The large and rapid Ca^2+^ influx through NMDARs can be cytotoxic if channel opening persists and is not regulated. Therefore, NMDARs are negatively regulated by intracellular Ca^2+^ elevation through a process known as either Ca^2+^-dependent inactivation (CDI) or as Ca^2+^-dependent desensitization (CDD), which is the term used in this review [32,33,34,35,36]. The term “desensitization” is more accurate than “inactivation” because the Ca^2+^-dependent inactivation of NMDAR activity is saturable and manifests as a fast-macroscopic desensitization [37]. CDD of NMDARs requires direct binding of CaM to the intracellular region of GluN1 [37,38] and GluN2A [12]. Previous studies revealed two intracellular CaM binding sites in GluN1: residues 841–865 (called GluN1-C0, Figure 1A,C,D) [38] and residues 875–898 (called GluN1-C1, Figure 1A,C,D) [19]. CaM binding to GluN1-C0 was demonstrated previously to be essential for CDD [33,38] and was suggested to promote dimerization of GluN1 [39]. By contrast, the GluN1-C1 site likely does not contribute to CDD [38] but rather it controls GluN1 binding to the cytoskeleton and its trafficking [19,40]. A single CaM binding site is also located in GluN2A (residues 1004–1023, called GluN2A-C0, Figure 1B–D) [18]. A crystal structure of CaM bound to the GluN1-C1 has been reported [19], along with two recent NMR structures of Ca^2+^-CaM bound separately to GluN1-C0 and GluN2A-C0 [12]. Mutations in NMDARs or CaM that cause defects in the Ca^2+^-dependent regulation of NMDAR channel function are associated with a spectrum of neurological diseases and neuropsychiatric disorders [41,42,43,44]. Elucidating NMDAR interactions with CaM may help improve our understanding of Ca^2+^-dependent channel regulation and provide a basis for treating neuronal diseases.

**Figure 3:**
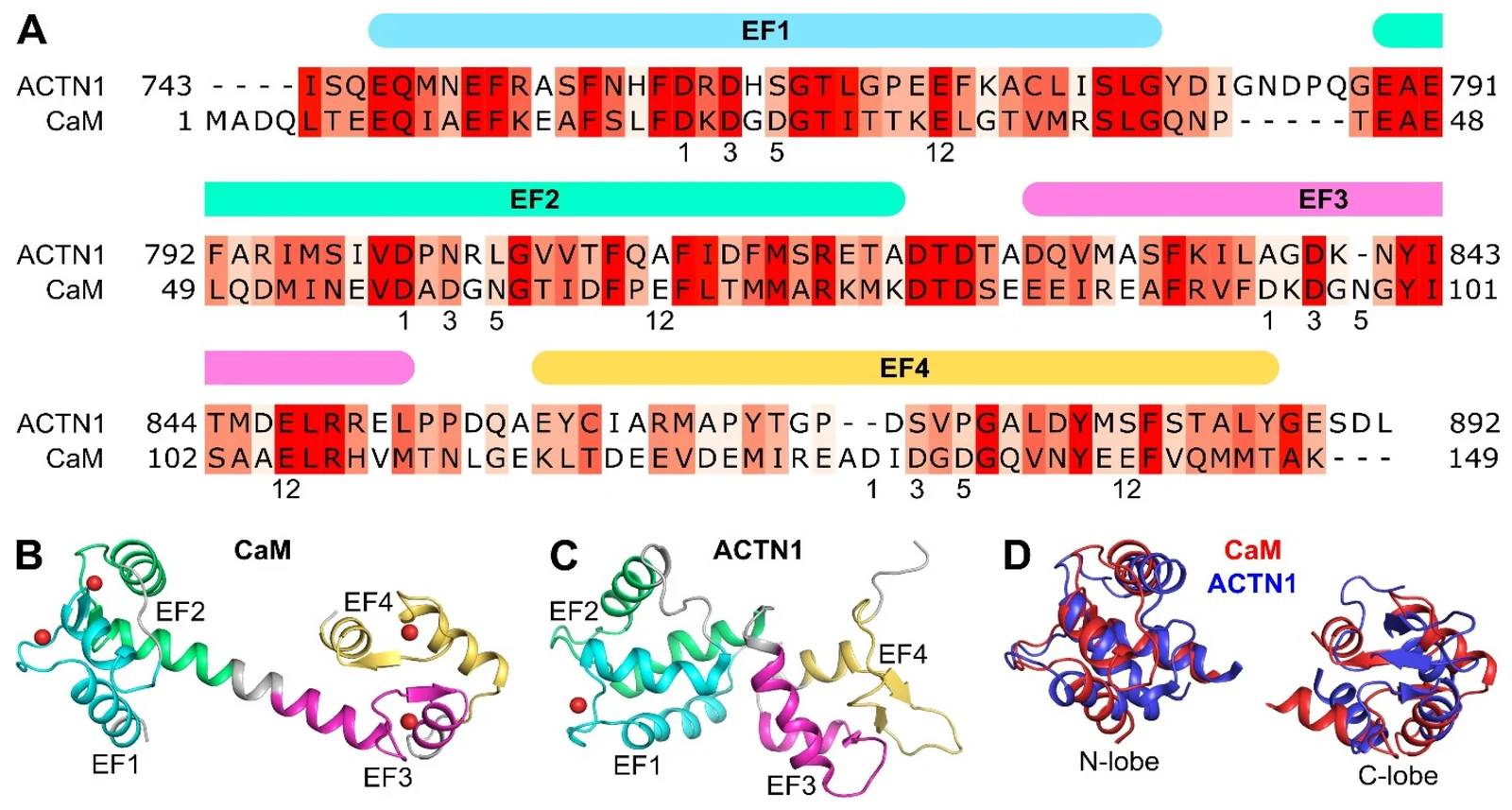
Sequence and structural comparison of CaM and ACTN1. (**A**) Amino acid sequence alignment of CaM and ACTN1. Sequence conservation is indicated by a red gradient, where dark red signifies highly conserved residues and light red represents areas of low conservation. Crystal structures of (**B**) Ca^2+^-CaM (PDB ID: 1CLL [46]) and (**C**) Ca^2+^-bound ACTN1 (PDB ID: 2N8Y [47]). The Ca^2+^ bound to EF1 in ACTN1 has a dissociation constant of ~40 μM [47] and is probably not physiologically relevant. The EF-hand motifs are shaded in color (EF1: cyan, EF2: green, EF3: magenta, and EF4: yellow). The four EF-hands are grouped into two domains: N-lobe (EF1 and EF2) and C-lobe (EF3 and EF4). Each EF-hand contains a 12-residue Ca^2+^-binding loop with conserved acidic residues marked at the 1, 3, 5, and 12 positions that chelate the bound Ca^2+^. Ca^2+^ ions are represented in red spheres. (**D**) Structural comparison of the N-lobe and C-lobe of CaM (red) and ACTN1 (blue).

CaM is a 16.7 kDa Ca^2+^ sensor protein that contains four EF-hand Ca^2+^ binding motifs (EF1 in cyan, EF2 in green, EF3 in magenta, and EF4 in yellow; Figure 3A,B). The 12-residue Ca^2+^-binding loop in each EF-hand contains conserved acidic residues at loop positions 1, 3, 5 and 12 (labeled underneath the sequence in Figure 3A) whose carboxylate side chains chelate the bound Ca^2+^. The four EF-hands are grouped into two domains: EF1 and EF2 interact structurally to form the N-lobe (residues 5–77 in Figure 3B), whereas EF3 and EF4 combine to form the C-lobe (residues 82–102 in Figure 3B). The binding of Ca^2+^ to each EF-hand promotes a conformational change, which causes the exposure of a hydrophobic groove in each lobe that binds to hydrophobic sites (typically aliphatic helices) in target binding proteins [45]. The recent NMR structure of Ca^2+^-CaM bound to the GluN1-C0 peptide [12] reveals that both CaM lobes bind to opposite sides of the GluN1-C0 helix. Key hydrophobic residues in GluN1 (M848 and F852) structurally contact the hydrophobic groove in the CaM C-lobe, whereas GluN1 residues (V855 and W858) contact the hydrophobic groove in the CaM N-lobe. The NMR structure of Ca^2+^-CaM bound to the GluN2A-C0 peptide [12] reveals that only the CaM C-lobe binds to the GluN2A-C0 helix in which GluN2A residues (W1014 and V1018) structurally contact the hydrophobic groove in the CaM C-lobe. The GluN1 point mutations (F852E and W858E) and GluN2A mutation (W1014E) each disrupt electrophysiologically measured CDD [12], suggesting CaM interaction with these residues contributes to CDD.

The Ca^2+^-free form of CaM (called apo-CaM) has been suggested to bind to GluN1 [32,33] and may play a role in NMDAR channel modulation. Surprisingly, apo-CaM binding to the GluN1-C0 or GluN2A-C0 peptide could not be detected by NMR titrations or by isothermal titration calorimetry (ITC) [12]. Therefore, in this review, we prefer to not speculate about a functional role for apo-CaM binding to NMDAR and will not present a model of the basal state. The apo-CaM binding to the GluN1-C0 peptide detected by fluorescence polarization studies [32] might be an artifact of the attached fluorescent probe in these studies, because the GluN1-C0 peptide (without a fluorescent tag) did not exhibit any binding to apo-CaM in either the NMR or ITC studies. The lack of any detectable heat signal in the ITC titration with apo-CaM might indicate a zero-enthalpy change (ΔH = 0) for apo-CaM binding to the GluN1-C0 peptide rather than zero binding. The lack of any NMR spectral change in the NMR titration with apo-CaM might be explained if the GluN1-C0 peptide binding does not cause a detectable structural change in apo-CaM. Future studies on EF-hand mutants of CaM (called CaM^12^, CaM^34^ and CaM^1234^ as described by [48]) that abolish Ca^2+^ binding to the particular EF-hands are needed to more rigorously probe the functional effects of Ca^2+^ binding. In particular, the CaM^1234^ mutant (that disables Ca^2+^ binding to all four EF-hands) should be studied to assess whether apo-CaM can pre-associate with NMDAR under basal conditions and whether apo-CaM binding is physiologically relevant. Alternatively, a half-calcified form of CaM (Ca^2+^ bound to EF3 and EF4 and not bound to EF1 and EF2) might pre-associate with NMDAR under resting Ca^2+^ conditions ([Ca^2+^] = 100 nM). Since the Ca^2+^-bound CaM C-lobe binds to the GluN1-C0 peptide with a dissociation constant of 100 nM [12], this implies that the CaM C-lobe bound to the C0 peptide should have an apparent Ca^2+^ affinity in the nanomolar range, suggesting that a significant fraction of the CaM C-lobe bound to NMDAR may have Ca^2+^ bound under basal conditions. This is analogous to the recent finding that L-type voltage-gated Ca^2+^ channels (Ca_V_1.2) are likely pre-associated with half-calcified CaM rather than apo-CaM [48].

## 3. GluN2 Subtype-Specific Ca^2+^-Dependent Desensitization of NMDARs

**Figure 4:**
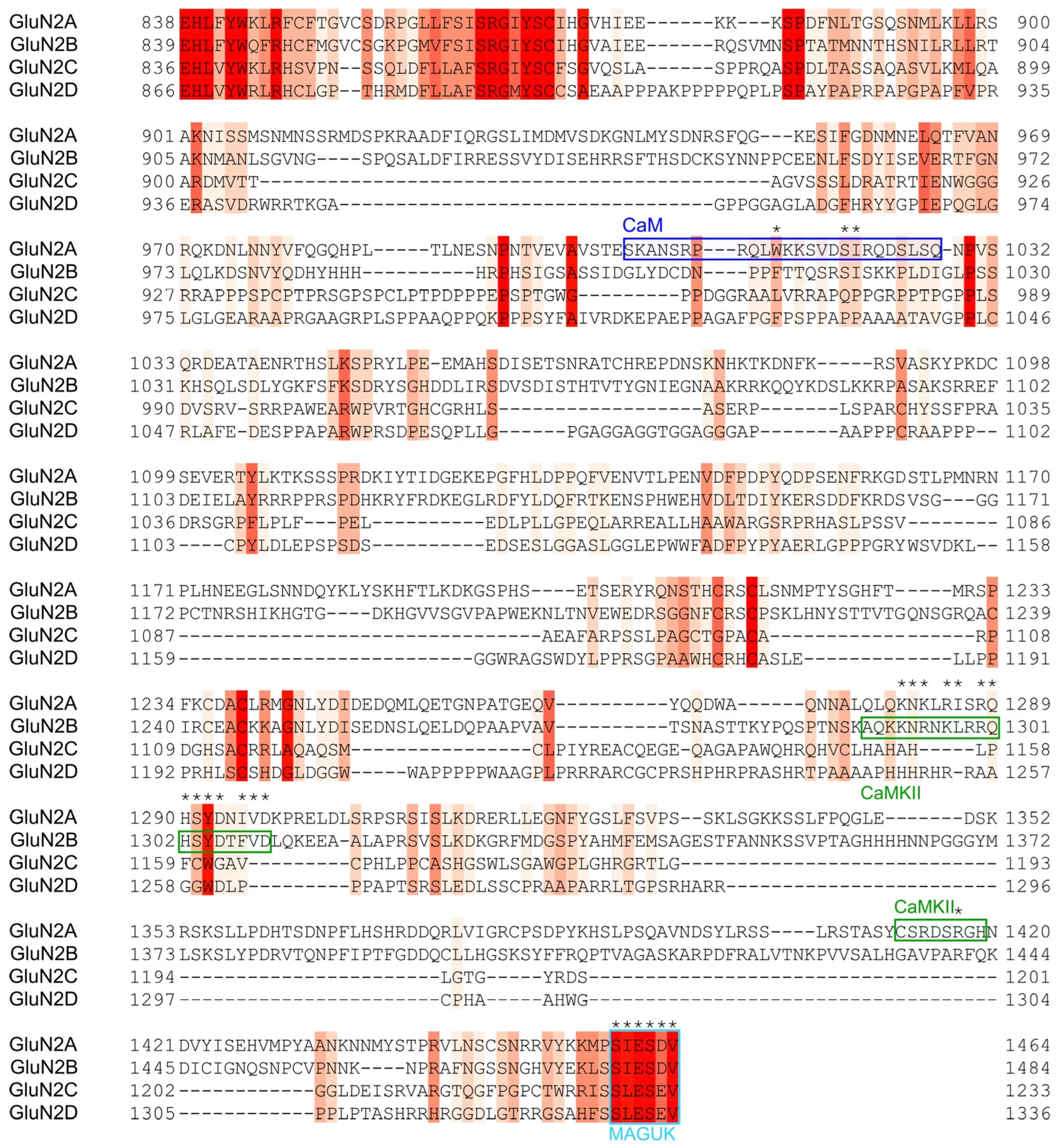
Sequence alignment of human GluN2A-D CTD. Sequence conservation is shown as a red color gradient, with dark red representing highly conserved residues and light red indicating low sequence conservation. Key interaction sites are highlighted with colored boxes: the CaM-binding site is shown in blue, the CaMKII-binding site in green, and the MAGUK-binding site in cyan. Conserved residues within the binding sites are highlighted with asterisks.

The magnitude of CDD in NMDARs differs markedly among NMDARs containing distinct GluN2 subtypes [34]. GluN2A-containing receptors (NMDAR heterotetramer with two GluN1 and two GluN2A, called GluN1/2A) exhibit the most pronounced desensitization, whereas GluN1/2B displays intermediate desensitization and GluN1/2C and GluN1/2D exhibit the weakest desensitization [49,50]. These differences are thought to arise from subtype-specific variations in channel gating, open probability, and allosteric coupling that govern receptor activation and desensitization. Although the four GluN2 subtypes differ in length, sequence alignments reveal that the ATD, LBD, and TMD are highly conserved (>50% sequence identity), in contrast to CTD (<35% identity). Sequence alignment of the human GluN2A–D CTDs further reveals that GluN2A and GluN2B share approximately 34% sequence identity, whereas GluN2C and GluN2D share approximately 30% sequence identity, highlighting the substantial sequence divergence within this regulatory domain (Figure 4). This divergence in the GluN2 CTDs enables interactions with numerous regulatory proteins, including CaM, Ca^2+^-CaM-dependent protein kinase II (CaMKII), protein kinase A (PKA), protein kinase C (PKC), casein kinase (CK), protein tyrosine kinases (Fyn and Src), and membrane-associated guanylate kinases (MAGUKs), and these interactions play critical roles in regulating NMDAR function [51]. Although multiple mechanisms contribute to subtype-specific gating and desensitization, this review focuses on the CaM-dependent CDD in NMDARs containing different GluN2 subtypes A–D. While the primary Ca^2+^-CaM binding site responsible for CDD is located in the C0 region of the GluN1 and GluN2A subunits [12], no well-established, functional CaM binding site has been identified in GluN2B, GluN2C, or GluN2D. Nevertheless, sequence alignment of the GluN2 CTD reveals that the GluN2A C0 region shares moderate sequence similarity with GluN2B (Figure 4). Notably, three critical CaM-binding residues identified in GluN2A (W1014, S1020, and I1021) are conserved in GluN2B (Figure 4, blue box, asterisks). This sequence conservation suggests a potential CaM-binding site in GluN2B that may contribute to the CDD observed in GluN1/2B [50]. Future experimental validation is required to determine whether CaM binding to this putative CaM-binding site in GluN2B is essential for CDD observed for GluN1/2B. In contrast, GluN2C and GluN2D exhibit low sequence conservation in the CaM-binding region and are predicted to not bind to CaM. In particular, the critical CaM-binding residues in GluN2A (W1014, S1020, and I1021) are not conserved in GluN2C and GluN2D. We propose that the relatively weak CDD observed in GluN1/2C and GluN1/2D may be caused by a lack of CaM binding to either GluN2C or GluN2D.

## 4. NMDAR Channel Activation by ACTN1

The cytoskeletal proteins, ACTN1 and ACTN2 (100 kDa), both contain an N-terminal actin binding domain, four spectrin coiled-coil repeats, and C-terminal EF-hand domain [52]. The EF-hand domain of ACTN1 (Figure 3A,C) was shown previously to bind to GluN1-C0 [20,22]. Also, ACTN2 binding to NMDAR has been suggested to increase channel open probability [21,53]. The EF-hand domain in ACTN1 has mutations to conserved acidic residues in the EF-hand Ca^2+^-binding loops (see S763 in EF1, L804 and A811 in EF2, A837 in EF3, and S872, P874, and S881 in EF4; see Figure 3A), which prevent Ca^2+^ binding in the physiological range. Therefore, ACTN1 binds weakly to Ca^2+^ in the high micromolar range and is thought to not function as a physiological Ca^2+^ sensor [47,54,55,56]. Previous studies have shown that, under Ca^2+^-free conditions, ACTN1 and ACTN2 both compete with CaM for binding to GluN1-C0 and prevent NMDAR channel desensitization (see Displacement Model below), whereas elevated intracellular Ca^2+^ levels promote CaM binding, which displaces ACTN from the same binding site [22,57]. A similar competitive binding mechanism has been observed in the Ca_V_1.2 L-type voltage-gated Ca^2+^ channel, where the Ca^2+^-free EF-hand domain of ACTN1 competes with CaM for binding to the IQ motif, and ACTN1 binding to the IQ causes an increase in Ca_V_1.2 channel open probability [55]. In this review, we suggest that the Ca^2+^-free EF-hand domain of ACTN1 binding to GluN1-C0 may stabilize the NMDAR channel in the ligand-bound open state at low cytosolic Ca^2+^ levels, in contrast to Ca^2+^-CaM binding to GluN1-C0 and GluN2A-C0 that stabilizes NMDAR in the ligand-bound desensitized state [12]. In essence, we suggest that ACTN1 and CaM work together to cause Ca^2+^-dependent NMDAR channel regulation: ACTN1 binding to NMDAR causes channel activation at low Ca^2+^ levels in contrast to Ca^2+^-CaM binding that displaces ACTN1 and causes channel desensitization at high cytosolic Ca^2+^ levels.

In this review, we focused on the structural modeling of ACTN1 binding to NMDAR, because a recent NMR study has characterized the structure of ACTN1 bound to the GluN1-C0 peptide [20]. Also, a crystal structure is known for ACTN1 [52], and NMR structures are known for the metal-free EF-hand domain of ACTN1 [20,47,55] and ACTN2 [54]. Importantly, the affinity of the GluN1-C0 peptide is tenfold higher for ACTN1 compared to ACTN2 [22]. The ACTN1 EF-hand region is comprised of separate N-lobe and C-lobe domains that are structurally similar to those of CaM (Figure 3D). The ACTN1 C-lobe was shown to interact structurally with the IQ motif in Ca_V_1.2 [55] and GluN1-C0 [20]. These ACTN1 structures will be combined with the recent cryo-EM structure of NMDAR in the open channel state [28] to construct a structural model of ACTN1 bound to NMDAR as described below. We believe that ACTN1 and ACTN2 are structurally similar based on the high sequence similarity for the residues that contact GluN1-C0. However, it is important to point out that the physiology of NMDAR regulation is better known for ACTN2 rather than ACTN1, and it is possible that ACTN1 and ACTN2 may have different roles in regulating channel function. Future electrophysiology studies are needed to better characterize NMDAR channel regulation by ACTN1.

## 5. Displacement Mechanism of NMDAR Channel Modulation

**Figure 5:**
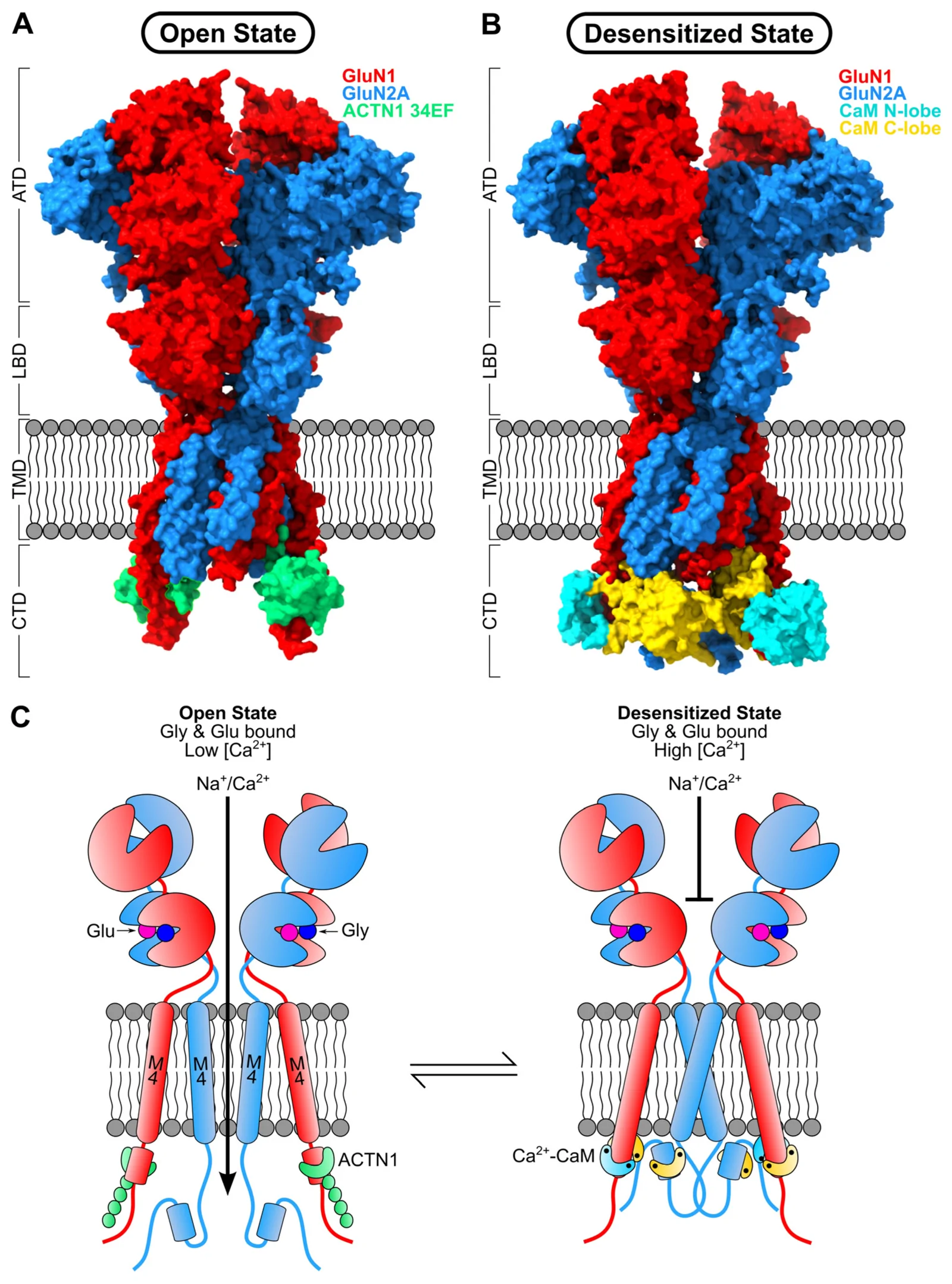
Hypothetical structural mechanism of NMDAR channel regulation by ACTN1 and CaM. (**A**) Surface representation of the NMDAR tetramer model (GluN1 in red and GluN2A in blue) in the open channel state bound to two ACTN1 molecules (green). This model was constructed using AlphaFold3 [58] and contained two GluN1 subunits (residues K25–R865), two GluN2A subunits (residues L34–Y842), and two ACTN1 C-lobes (residues D822–L892), followed by energy minimization under vacuum conditions using MD simulations as described previously [74]. (**B**) Surface representation of the NMDAR tetramer model (GluN1 in red and GluN2A in blue) in the desensitized channel state bound to four Ca^2+^-CaM molecules (N-lobe in cyan and C-lobe in yellow). The CaM binding stoichiometry of 4 CaM bound per tetramer is assumed in the modeling and is based on previous binding studies [12] that determined each C0 peptide binds to one CaM molecule, which suggests that 4 CaM bind per tetramer because each tetramer (GluN1/2A) has four C0 sites. The structural model of the NMDAR tetramer featuring structured C0 sites was generated using AlphaFold3 [58]. The details of the modeling methods were described recently by [12]. The tetramer model contains two GluN1 subunits (residues K25–R865) and two GluN2A subunits (residues L34–Q1023). NMR structures of Ca^2+^-CaM bound to GluN1-C0 were superimposed onto the GluN1-C0 sites within the NMDAR tetramer model. NMR structures of the Ca^2+^-CaM C-lobe bound to GluN2A-C0 were manually docked into the NMDAR tetramer. The NMDAR–CaM complex was energy-minimized using molecular dynamics (MD) simulations. Atomic coordinates of the structural models will be made available upon request. (**C**) Schematic summary of conformational changes of NMDAR that occur during the transition from the agonist-bound open channel state (**left** panel) to the Ca^2+^-induced and agonist-bound desensitized state (**right** panel). Under low Ca^2+^ concentrations, GluN1-C0 is anchored to ACTN1 C-lobe (green), which increases the channel open probability of the NMDAR. The vertical arrow indicates Ca^2+^ influx through the open channel. Under high Ca^2+^ levels, four Ca^2+^-CaM molecules (N-lobe in cyan; C-lobe in yellow) bind to the C0 domains of GluN1 and GluN2A, leading to channel desensitization. The vertical bar indicates the channel pore is closed. Bound agonists (Glu and Gly) are represented as magenta and blue spheres.

We propose a “displacement” mechanism for CDD in which ACTN1 binding to GluN1-C0 (in the open channel state) is displaced by Ca^2+^-CaM binding, which promotes channel desensitization (Figure 5). At low cytosolic Ca^2+^ levels, we suggest that ACTN1 binding to GluN1-C0 stabilizes the channel open state. By contrast, at high Ca^2+^ levels, Ca^2+^-CaM displaces ACTN1 and binds to both GluN1-C0 and GluN2A-C0 to stabilize the desensitized channel state. A structural model of the NMDAR desensitized channel tetramer bound to Ca^2+^-CaM was constructed using AlphaFold3 [58] (Figure 5B,C right panel). In this model, each GluN1 M4 helix extends continuously into the C0 helix, thus forming one long continuous helix that connects M4 and C0. The NMR structure of Ca^2+^-CaM bound to GluN1-C0 [12] was superimposed onto each GluN1 C0 helix in the tetramer model. NMR structures of Ca^2+^-CaM bound to GluN2A-C0 [12] were manually docked into the NMDAR tetramer (Figure 5B). Our model of the desensitized channel tetramer suggests that Ca^2+^-CaM binds to helical structures in GluN1-C0 and GluN2A-C0 that come together to form a concentric ring-like structure located underneath the channel pore, which may serve to allosterically stabilize the desensitized channel and facilitate closure of the pore (see cyan and yellow region in Figure 5B). At low Ca^2+^ levels, the competitive binding of ACTN1 displaces CaM from GluN1-C0, and Ca^2+^-free CaM dissociates from GluN2A-C0 (Figure 5A). This removal of CaM from NMDAR and subsequent binding of ACTN1 is predicted to destabilize the ring-like concentric arrangement of the GluN1-C0 and GluN2A-C0 helices underneath the channel pore. The Ca^2+^-sensitive dismantling of the concentric complex of GluN1-C0 and GluN2A-C0 is suggested here to facilitate a conformational change in the M4 helix of both GluN1 and GluN2A that promotes channel opening at low Ca^2+^ levels (see black arrow in Figure 5C). A structural model of the NMDAR open channel tetramer bound to the ACTN1 C-lobe (Figure 5A,C left panel) was generated using AlphaFold3 [58]. Our model of the channel open state suggests that the C-lobe of ACTN1 binds to the helical structure of GluN1-C0 as suggested recently by [20], and ACTN1 is not bound to GluN2A-C0. As a result, GluN2A-C0 becomes unstructured and no longer interacts with GluN1-C0, which dissociates the concentric complex of GluN1-C0/GluN2A-C0 and facilitates channel opening (Figure 5C). ACTN1 also serves to bridge NMDARs to the cytoskeleton, which enhances the cell surface expression of NMDARs [22,59]. This bridging of NMDARs to the cytoskeleton is mediated by ACTN1 in which its N-terminal domain (actin binding domain) connects to the actin cytoskeleton, while the other end of ACTN1 (C-terminal EF-hand region) binds to GluN1. ACTN1 also bridges Ca_V_1.2 to the cytoskeleton and enhances the Ca_V_1.2 cell surface expression in the brain and heart [55]. Future studies are needed to experimentally determine atomic-level structures of the NMDAR bound to both Ca^2+^-CaM and ACTN1 to more rigorously test the Ca^2+^-dependent conformational changes predicted by our model (Figure 5C).

Our model suggests that Ca^2+^ binding to CaM is essential for promoting CDD (Figure 5C). Thus, mutations in CaM that weaken or disable Ca^2+^ binding are expected to disrupt CDD, which could enable unregulated Ca^2+^ influx and possibly cause neuronal hyperexcitability. Calmodulinopathies are a class of diseases in which de novo mutations in CaM (D94A, D96G, N98S, E105A, D130A, D132G, D134H, and E141G) impair Ca^2+^ binding to the third or fourth EF-hands and promote fatal cardiac arrhythmias caused by impaired Ca^2+^-dependent inactivation of various Ca^2+^ channels (ryanodine receptor 2, RyR2 [60,61] and Ca_V_1.2 [62]). In particular, CaM mutations that disrupt inactivation of RyR2 cause a phenotype known as catecholaminergic polymorphic ventricular tachycardia (CPVT) [63], whereas the CaM mutations that disrupt CDI of Ca_V_1.2 cause a distinct phenotype known as long-QT syndrome (LQTS) [64]. Calmodulinopathies also cause epileptic seizures [65,66] and cognitive deficits [67]. The role of NMDARs in neuronal excitability implies that dysregulation of CDD may contribute to these pathologies. Indeed, weakened CaM binding to GluN1 has been observed in epileptic disorders [68]. Future studies are needed to explore whether any of the known CaM mutations (D94A, D96G, N98S, E105A, D130A, D132G, D134H, and E141G) might also disrupt CDD in NMDARs and whether this dysregulation of NMDARs might contribute to calmodulinopathies.

Our model suggests how CaM binding to NMDAR (Figure 5C, right panel) might control the sensitivity of the NMDAR channel blocker, memantine, which is clinically approved for treating Alzheimer’s Disease [69] and shows promise for treating Parkinson’s Disease [70]. A recent study revealed that inhibition of NMDARs by memantine increases with increasing intracellular Ca^2+^ concentration [71], and a separate study demonstrated that memantine binds to NMDAR (GluN1/2A) with about 3-fold higher affinity when the intracellular Ca^2+^ concentration is raised to ~5.0 μM in the presence of overexpressed and recombinant wildtype CaM but not in the presence of CaM^1234^ [72]. These results indicate that Ca^2+^-CaM binding to NMDAR is essential for achieving its high-affinity binding to memantine. The results also suggest that memantine selectively binds to the desensitized channel state bound to Ca^2+^-CaM (Figure 5C, right panel). We suggest that Ca^2+^-CaM binding to C0 may allosterically stabilize the memantine binding site in the channel pore as observed for GluN1/2B [73]. The preferential binding of memantine to the desensitized channel bound to Ca^2+^-CaM selectively inhibits a subpopulation of NMDARs that promote excitotoxicity, which may explain why memantine has such high clinical safety [71]. Future studies are needed to determine the cryo-EM structure of GluN1/2A bound to memantine and to elucidate how memantine can selectively stabilize the desensitized channel bound to Ca^2+^-CaM.

## 6. Conclusions

NMDARs are Ca^2+^-permeable ionotropic glutamate receptors that serve as important Ca^2+^ channels in the brain and have critical roles in neural development, synaptic plasticity, learning, and memory. Channel opening causes Ca^2+^ influx that activates a range of Ca^2+^-dependent cellular processes. NMDAR channel activity is finely regulated by α-actinin, which promotes channel opening, and by CaM, which mediates Ca^2+^-dependent channel desensitization (CDD). We propose a “displacement” mechanism for CDD in which ACTN1 binding to NMDAR stabilizes the channel open state under basal conditions (at low cytosolic Ca^2+^ levels). At high Ca^2+^ levels (following neuronal stimulation), ACTN1 is displaced by Ca^2+^-CaM binding to NMDAR, which stabilizes the desensitized channel state. Our model (Figure 5C) suggests that Ca^2+^-CaM binding to NMDAR promotes the formation of helical structures in GluN1-C0 and GluN2A-C0 that come together to form a concentric ring-like complex located underneath the channel pore that may serve to allosterically stabilize the desensitized channel and facilitate closure of the pore.

## Data Availability

Atomic coordinates of the structures in Figure 2 and Figure 3 are openly available in the Protein Data Bank (https://www.rcsb.org). Atomic coordinates of the structural models in Figure 5 will be made available from the authors upon request.

## Abbreviations

The following abbreviations are used in this manuscript:

ACTN	α-actinin
ACTN1	α-actinin-1
ACTN2	α-actinin-2
CaM	calmodulin
CDD	Ca^2+^-dependent desensitization
ATD	Amino-terminal domain
LBD	Ligand binding domain
TMD	Transmembrane domain
CTD	Carboxy-terminal domain
NMDAR	N-methyl D-aspartate receptor
